# Prevalence of co-trimoxazole resistance among HIV-infected individuals in Ethiopia: a systematic review and meta-analysis

**DOI:** 10.3389/fmed.2024.1418954

**Published:** 2024-07-11

**Authors:** Muluneh Assefa, Getu Girmay

**Affiliations:** ^1^Department of Medical Microbiology, School of Biomedical and Laboratory Sciences, College of Medicine and Health Sciences, University of Gondar, Gondar, Ethiopia; ^2^Department of Immunology and Molecular Biology, School of Biomedical and Laboratory Sciences, College of Medicine and Health Sciences, University of Gondar, Gondar, Ethiopia

**Keywords:** co-trimoxazole resistance, human immunodeficiency virus, systematic review, meta-analysis, Ethiopia

## Abstract

**Background:**

Co-trimoxazole is used as a prophylaxis for human immunodeficiency virus (HIV) patients to prevent opportunistic infections. Its widespread use results in the emergence of co-trimoxazole resistance, which is a significant problem. This systematic review and meta-analysis determined the pooled prevalence of co-trimoxazole resistance among HIV-infected individuals in Ethiopia.

**Methods:**

The Preferred Reporting Items for Systematic Reviews and Meta-Analyses (PRISMA) guideline was applied to report this study. The protocol has been registered in the International Prospective Register of Systematic Reviews (PROSPERO) under the assigned number CRD42024532240. Article search was performed using electronic databases such as PubMed, Medline, EMBASE, Google Scholar, Hinari, Web of Science, Science Direct, and African Journals Online. Data were extracted using a Microsoft Excel spreadsheet and analyzed using STATA version 11.0 software. A random-effects model was used to estimate the pooled effect size of co-trimoxazole resistance across studies with a 95% confidence interval. The heterogeneity was checked using I^2^ statistic. The presence of publication bias was determined using a funnel plot and Egger’s test with a *p*-value <0.05 evidence of statistically significant bias. Subgroup and sensitivity analyses were performed.

**Results:**

Twenty-two studies with 5,788 HIV-infected individuals were included. The pooled prevalence of co-trimoxazole resistance was 61.73% (95% CI: 53.10–70.37%), with heterogeneity (I^2^ = 87.7%) and statistical significance (*p* < 0.001). A higher co-trimoxazole resistance was observed in HIV-infected individuals with urinary tract infection; 82.10% (95% CI: 75.03–89.17%). Among the bacterial spp., higher resistance to co-trimoxazole was observed in *Escherichia coli*; 70.86% (95% CI: 53.44–88.27%) followed by *Salmonella* spp.; 67.66% (95% CI: 41.51–93.81%) and *Proteus* spp.; 66.23% (95% CI: 34.65–97.82%).

**Conclusion:**

There is a higher prevalence of co-trimoxazole resistance in HIV-infected individuals in Ethiopia. This alarms WHO’s recommendation of co-trimoxazole prophylaxis guidelines to review and update it. Additionally, a nationwide assessment of co-trimoxazole resistance in Ethiopia as a whole is required.

**Systematic review registration**: identifier: CRD42024532240.

## Introduction

Human immunodeficiency virus (HIV), which later causes acquired immunodeficiency syndrome (AIDS), is one of the global health threats (1). According to the Joint United Nations Programme on HIV/AIDS, 2022 Global HIV and AIDS Statistics fact sheet, 84.2 million people have become infected with HIV since the HIV epidemic began, and 40.1 million have died from AIDS-related illnesses. Besides, around 650,000 people died from AIDS-related illnesses worldwide in 2021 (2). Ethiopia has made remarkable strides in controlling the HIV/AIDS epidemic over the past decade. However, the prevalence remains relatively high in urban areas where estimates indicate a 3 % rate compared to under 1 % nationally (3).

Co-trimoxazole, a combination of trimethoprim and sulfamethoxazole antibiotics, is recommended as a prophylaxis for HIV-infected individuals to prevent opportunistic infections; however, the indication may vary according to CD4 cell count, age, geographical setting, and prior medical history (4, 5). The use of co-trimoxazole prophylaxis in low-income settings is recommended by the World Health Organization (WHO) for all children with HIV and for adults with advanced HIV infection or who are living in areas where bacterial infections are prevalent, irrespective of antiretroviral therapy (ART) (4, 6).

Previously, research had shown that co-trimoxazole prophylaxis decreases morbidity and death in HIV-infected individuals (7). However, the current scenario might be different with over 80% of bacteria causing opportunistic infections are resistant to co-trimoxazole (8). The widespread use of co-trimoxazole prophylaxis in HIV patients is thought to be the cause of an increasing burden of antimicrobial resistance throughout the AIDS era. The emergence of co-trimoxazole resistance among HIV-infected individuals has become a significant problem (8). A study in Tanzania reported a 75% bacterial resistance to co-trimoxazole from HIV patients and 81.3% resistance in patients with prophylaxis (8).

In Ethiopia, different individual studies were conducted on the pattern of co-trimoxazole resistance to bacterial infections or colonizations in HIV-infected individuals. This systematic review and meta-analysis report is necessary to provide valuable data on the status of co-trimoxazole resistance among HIV-infected individuals in Ethiopia, which is crucial for revising the recent guideline of co-trimoxazole prophylaxis. Therefore, this systematic review and meta-analysis determined the pooled prevalence of co-trimoxazole resistance among HIV-infected individuals in Ethiopia.

## Methods

### Study design and protocol registration

This systematic review and meta-analysis were performed as per the Preferred Reporting Items for Systematic Reviews and Meta-Analyses (PRISMA) guidelines (9) (Supplementary Table S1). The protocol has been registered in the International Prospective Register of Systematic Reviews (PROSPERO) under the assigned number CRD42024532240.

### Database search strategy

The co-trimoxazole resistance in bacterial pathogens that infect or colonize HIV-positive people was the main focus of this meta-analysis. The appropriateness of the included studies for this meta-analysis was assessed using the COCOPOP (Condition, Context, and Population) paradigm. Ethiopia served as the context (CO), HIV-infected individuals as the population (POP), and the study’s condition (CO) was the prevalence of co-trimoxazole resistance. Studies published before 2024 were included in the search, and it was last conducted until March 25, 2024. Electronic databases such as PubMed, Medline, EMBASE, Google Scholar, Hinari, Web of Science, Science Direct, and African Journals Online were searched to identify articles reporting the prevalence of co-trimoxazole resistance among HIV-infected individuals in Ethiopia. We used search terms alone and in combination with Boolean operators such as “OR” or “AND.” An example of a PubMed search strategy used was as follows: (((((bacteria) OR (infection)) OR (colonization))) AND ((((((antimicrobial resistance) OR (antibiotic resistance)) OR (co-trimoxazole resistance)) OR (trimethoprim-sulfamethoxazole resistance)) OR (antibiogram))) AND ((((human immunodeficiency virus) OR (immunocompromised)) OR (HIV/AIDS)) OR (HIV))) AND (Ethiopia). A manual search was conducted for relevant papers in the references of the included studies and other reviews. The articles retrieved were imported into EndNote X9 bibliographic software manager (Clarivate Analytics, Philadelphia, PA, United States).

### Outcome of interest

The main finding in this systematic review and meta-analysis was the national assessment of the pooled prevalence of co-trimoxazole resistance among HIV-infected individuals. Co-trimoxazole resistance based on the type of bacteria spp. was the second outcome of this study.

### Study selection and eligibility criteria

Two independent reviewers (MA and GG) screened the titles and abstracts of the identified studies to determine their eligibility. Full-text articles were then assessed for eligibility, and any disagreements between the reviewers were resolved through discussion. Articles published in the English language with a cross-sectional study design that included the prevalence of co-trimoxazole resistance in HIV-infected individuals in the results, studies conducted in Ethiopia and without limit on specimen type, type of infection or colonization, and study period. Studies not reporting co-trimoxazole resistance profile, case reports, review articles, meta-analysis studies, conducted on tuberculosis, sexually transmitted infections (viral and protozoa), or parasites, studies not conducted in Ethiopia, and study populations other than HIV-infected individuals were excluded from the study.

### Quality assessment

After removing duplicated papers, all potentially eligible papers were reviewed. Full-text papers were retrieved for review and relevant information was extracted. The Joana Briggs Institute (JBI) critical appraisal checklist for prevalence study was used to assess the quality of included studies (10). Accordingly, articles with high and medium quality (fulfilling 50% of quality assessment) were included in the final analysis (Supplementary Table S2). Two independent authors (MA and GG) assessed the quality of the studies, and any disagreement was solved by discussion.

### Data extraction

Data were extracted from each study using the designed tool in Microsoft Excel 2019 (Microsoft Corp., Redmond, WA, United States) by two independent authors (MA and GG). Any ambiguity and difference during extraction were resolved through discussion. The data extracted from eligible studies were author name, year of publication, area in which the study was conducted, study design, type of sampling method, number of HIV-infected individuals, sex (male), age group, type of infection or colonization, number of total isolates, bacterial group as Gram-positive or Gram-negative, number of bacterial species, co-trimoxazole resistance, and co-trimoxazole prophylaxis.

### Data analysis and synthesis

The relevant data were extracted from the eligible studies into a Microsoft Excel spreadsheet and then exported to the STATA version 11.0 software for meta-analysis. The pooled prevalence of co-trimoxazole resistance and 95% confidence intervals were visually displayed using a forest plot. Subgroup analysis was performed based on region, sampling technique, year of study, type of infection or colonization, and bacterial group. The heterogeneity of the included studies was evaluated using the inverse variance index (I^2^ statistic). No, low, moderate, and high levels of heterogeneity were considered when the values of I^2^ were 0, 25, 50, and 75%, respectively (11). In all pooled analyses, heterogeneity resulting from differences in effects across different studies was accounted for using a random-effects model. A sensitivity analysis of each study’s impact on the overall prevalence was also conducted. Publication bias was statistically investigated using Egger’s test (12) and visual inspection of funnel plots. A *p*-value <0.05 in Egger’s test was considered as evidence of statistically significant publication bias.

## Results

### Search results

**Figure 1:**
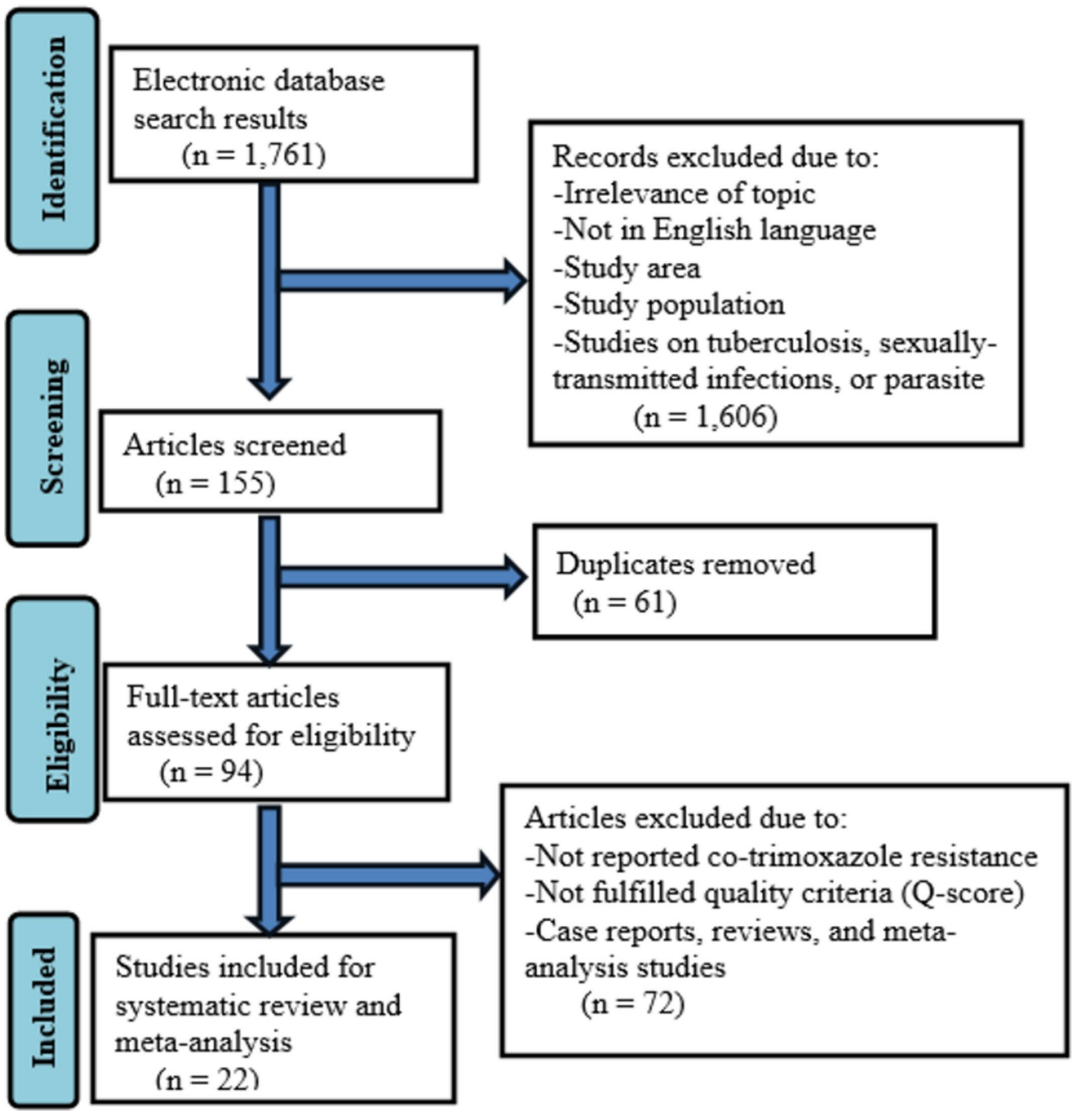
Flow diagram describing the selection of studies for the systematic review and meta-analysis on the prevalnce of co-trimoxazole resistance among HIV-infected individuals in Ethiopia.

A total of 1761records were retrieved during an electronic database search. The 155 were screened after removing records for several reasons such as studies with irrelevance of topics, not published in the English language, studies on tuberculosis, sexually transmitted infections, or intestinal parasites, studies not conducted in Ethiopia, and study populations other than HIV-infected individuals. Moreover, 61 duplicates were removed and 94 full-text articles were assessed for eligibility. Seventy-two full-text articles were excluded because of no co-trimoxazole resistance data, not fulfilling study quality criteria (Q-score), case reports, review articles, and meta-analysis studies. Finally, 22 studies were included in the systematic review and meta-analysis (Figure 1).

### Studies characteristics

A total of 22 studies (13–34) comprising 5788HIV-infected individuals were included in this systematic review and meta-analysis. All of these studies used a cross-sectional study design. Regarding the sampling technique, 11 studies used systematic random sampling, 8 studies used convenient sampling, and the remaining 3 studies did not report their sampling techniques. Studies included 9 from Amhara, 5 from South Ethiopia, 3 from Addis Ababa, 2 from Sidama, and 1 from (Oromia, Harari, and Tigray). The minimum and maximum numbers of study participants were 100 in Gondar (19) and 450 in Addis Ababa (16), respectively. Of the 5788HIV-infected individuals, 3,318 (57.3%) were females. The proportion of HIV-infected individuals on co-trimoxazole prophylaxis was not consistently reported in published articles; only 40.9% of studies have reported the status of prophylaxis. Studies reported that a total of 859 HIV-infected individuals were given the prophylaxis (Table 1).

**Table 1:** Summary of studies on the prevalence of co-trimoxazole resistance among HIV-infected individuals in Ethiopia.

Author, year	Study region	Study year	Sampling technique	HIV-infected individuals	COT prophylaxis	Sex (M)	Age group	Infection/ colonization	Bacterial group	COT resistance	Total isolates
Tilahun et al., 2023 (22)	Amhara	2021	Systematic random	378	193	187	≥10	Pneumonia	GPB & GNB	98	139
Adhanom et al., 2019 (23)	Tigray	2016	Not reported	252	Not reported	127	≥18	Pneumonia	GPB & GNB	40	106
Genetu & Zenebe, 2020 (26)	Amhara	2019	Convenient	163	Not reported	62	≥18	Pneumonia	GPB & GNB	46	65
Ayele et al., 2020 (18)	South Ethiopia	2019	Systematic random	180	65	84	≥15	Diarrhea	GNB	8	15
Alebachew et al., 2016 (19)	Amhara	2013	Convenient	100	Not reported	12	all age	Bloodstream infection	GPB & GNB	22	31
Tessema et al., 2020 (21)	Sidama	2018	Systematic random	224	40	93	≥18	Urinary tract infection	GPB & GNB	14	23
Abebe et al., 2014 (34)	Amhara	2013	Systematic random	113	Not reported	53	≥11	Colonization	GPB	45	103
Gebre et al., 2022 (27)	Addis Ababa	2016–18	Not reported	183	Not reported	90	<15	Colonization	GPB	12	50
Bayleyegn et al., 2021 (24)	Amhara	2020	Convenient	161	Not reported	77	<15	Colonization	GNB	81	186
Jemal et al., 2020 (20)	Amhara	2018	Convenient	384	Not reported	157	All age	Bloodstream infection	GPB & GNB	65	123
Manilal et al., 2019 (14)	South Ethiopia	2017	Systematic random	307	19	131	≥18	Colonization	GPB	25	64
Fenta et al., 2016 (16)	Addis Ababa	2015	Convenient	450	Not reported	141	≥18	Urinary tract infection	GPB & GNB	34	44
Simeneh et al., 2022 (17)	South Ethiopia	2021	Systematic random	251	14	114	≥18	Urinary tract infection	GPB & GNB	30	39
Muhaba et al., 2022 (25)	Amhara	2020	Systematic random	206	Not reported	77	All age	Colonization	GPB	50	58
Seid et al., 2020 (15)	South Ethiopia	2018	Systematic random	252	126	144	≥15	Colonization	GPB	24	34
Mulu et al., 2018 (13)	Amhara	2016–17	Convenient	300	Not reported	153	6–15	Colonization	GPB & GNB	85	162
Adisu et al., 2023 (28)	Oromia	2021	Convenient	351	Not reported	135	≥18	Colonization	GPB	10	21
Mitiku et al., 2023 (29)	South Ethiopia	2022	Systematic random	422	Not reported	204	≥18	Diarrhea	GNB	9	63
Tadesse et al., 2017 (30)	Addis Ababa	2013–14	Not reported	162	120	52	≥18	Diarrhea	GNB	22	24
Marami et al., 2019 (31)	Harari	2016	Systematic random	350	202	106	≥18	Urinary tract infection	GPB & GNB	55	63
Alemu et al., 2013 (32)	Amhara	2011–12	Systematic random	384	Not reported	168	≥18	Urinary tract infection	GPB & GNB	36	41
Kebede et al., 2017 (33)	Sidama	2016	Convenient	215	80	103	≥18	Diarrhea	GNB	20	29

HIV, human immunodeficiency virus; GPB, gram-positive bacteria; GNB, gram-negative bacteria; COT, co-trimoxazole; M, male.

### Pooled prevalence of co-trimoxazole resistance among HIV-infected individuals

**Figure 2:**
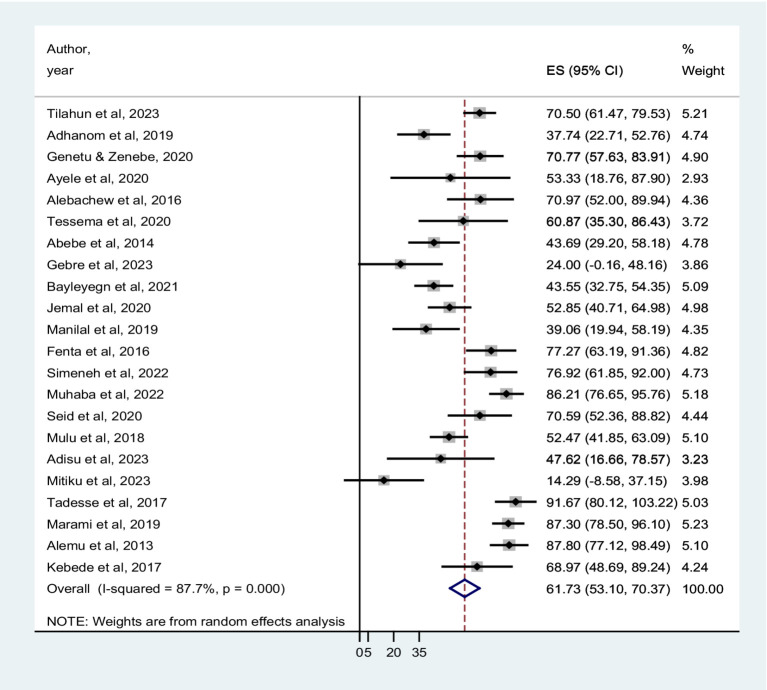
Forest plot showed the pooled prevalence of co-trimoxazole resistance among HIV-infected individuals in Ethiopia.

The prevalence of co-trimoxazole resistance reported by the studies ranged from 14.3% in Dilla (29) to 91.7% in Addis Ababa (30). Among 1,483 bacterial isolates identified from the eligible studies, 831 were co-trimoxazole-resistant (Table 1). Accordingly, the pooled prevalence of co-trimoxazole resistance was 61.73% (95% CI: 53.10–70.37%), with heterogeneity (I^2^ = 87.7%) and statistical significance (*p* < 0.001) (Figure 2).

### Subgroup analysis

**Figure 3:**
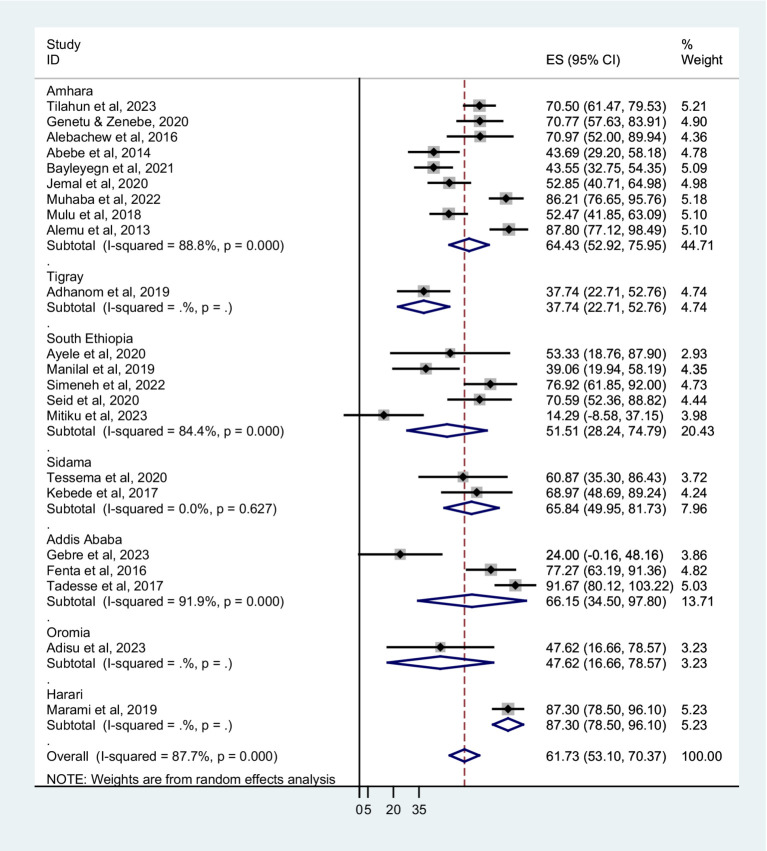
Forest plot showed subgroup analysis based on study region.

**Figure 4:**
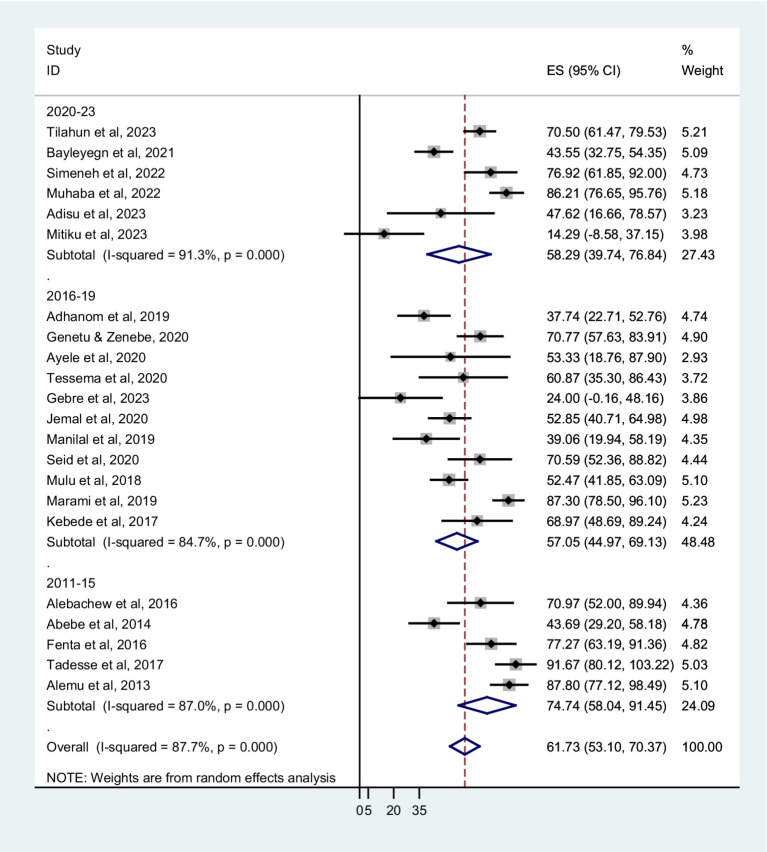
Forest plot showed subgroup analysis based on year of study.

The prevalence of co-trimoxazole resistance among regions of the studies performed, years of the study, sampling techniques, different types of infection or colonization, and bacterial groups were analyzed by subgroup analysis. The combined prevalence of co-trimoxazole resistance across the Ethiopian regions showed that the highest co-trimoxazole resistance was reported in Harari; 87.30% (95% CI: 78.50–96.10%), followed by Addis Ababa; 66.15% (95% CI: 34.50–97.80%), Sidama; 65.84% (95% CI: 49.95–81.73%), and Amhara; 64.43% (52.92–75.9%) (Figure 3). The pooled prevalence of co-trimoxazole resistance per year of study was as follows: 2011–15; 74.74% (95% CI: 58.04–91.45%), 2016–19; 57.05% (95% CI: 44.97–69.13%), and 2020–23; 58.29% (95% CI: 39.74–76.84%) (Figure 4).

**Figure 5:**
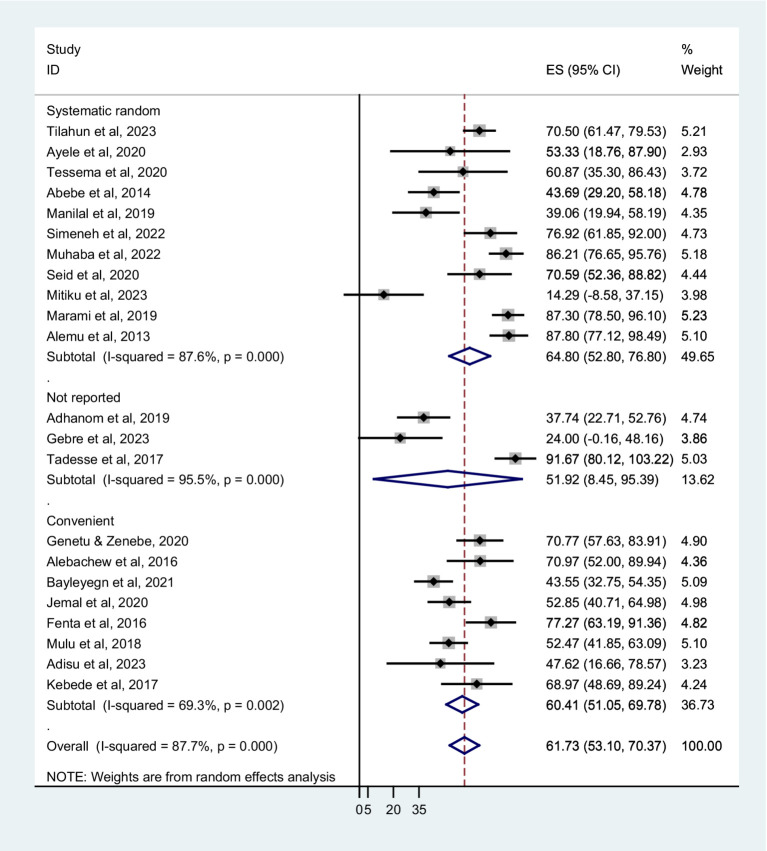
Forest plot showed subgroup analysis based on sampling technique.

**Figure 6:**
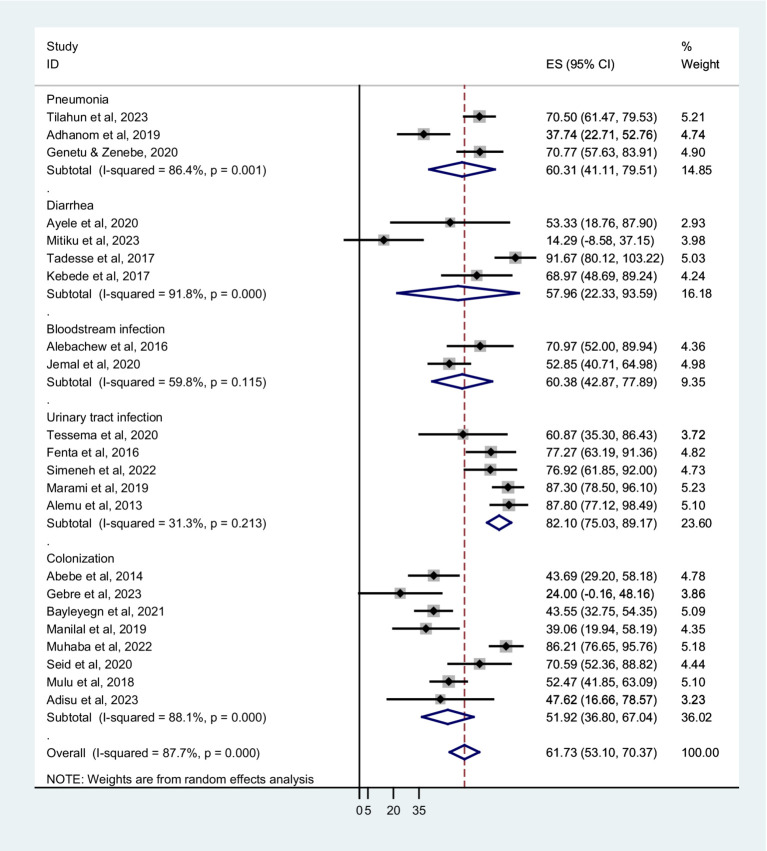
Forest plot showed subgroup analysis based on type of infection or colonization.

**Figure 7:**
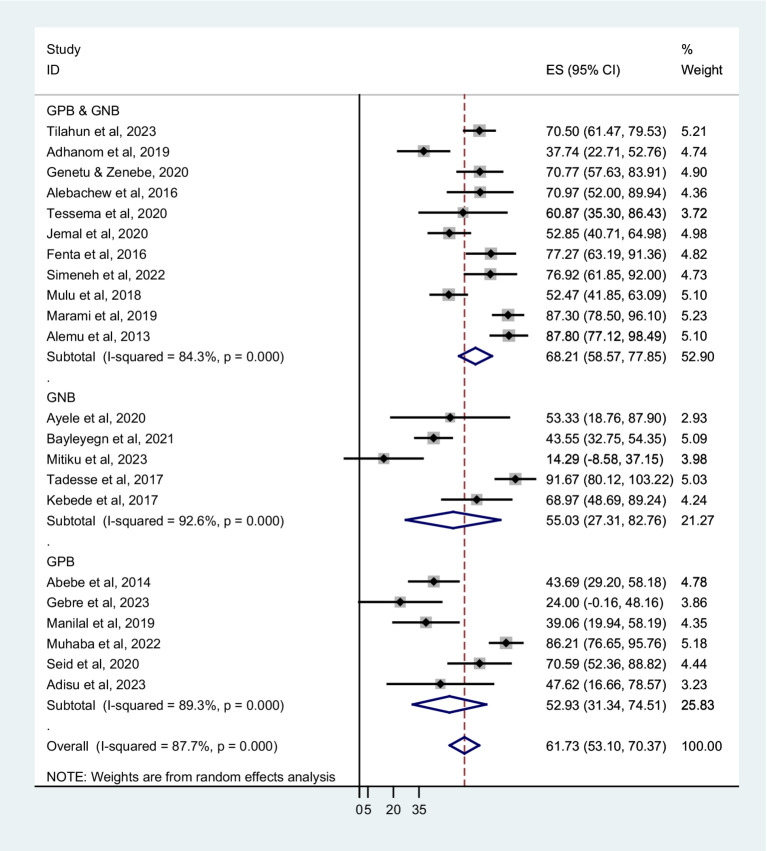
Forest plot showed subgroup analysis based on bacterial group.

Based on sampling techniques, the co-trimoxazole resistance was described as systematic random; 64.80% (95% CI: 52.80–76.80%), convenient sampling; 60.41% (95% CI: 51.05–69.78%), and studies have not reported the sampling method; 51.92% (95% CI: 8.45–95.39%) (Figure 5). According to the type of infection, a higher co-trimoxazole resistance was observed in HIV-infected individuals with urinary tract infection; 82.10% (95% CI: 75.03–89.17%), bloodstream infection; 60.38% (95% CI: 42.87–77.89%), and pneumonia; 60.31% (95% CI: 41.11–79.51%). The pooled prevalence of co-trimoxazole resistance in bacterial colonization was 51.92% (95% CI: 36.80–67.04%) (Figure 6). Furthermore, the pooled prevalence of co-trimoxazole resistance in HIV-infected individuals per bacterial group was high in studies identifying both Gram-positive and Gram-negative bacterial isolates; 68.21% (95% CI: 58.57–77.85%) (Figure 7).

### Pooled prevalence of co-trimoxazole resistance among bacterial species

Both Gram-positive and Gram-negative bacterial spp. that were reported by at least three studies were included in this meta-analysis. The pooled proportion of co-trimoxazole resistance for each bacterial spp. was computed from the total isolates tested for co-trimoxazole antibiotic. Among the bacterial spp., higher resistance to co-trimoxazole was observed in *Escherichia coli*; 70.86% (95% CI: 53.44–88.27%) followed by *Salmonella* spp.; 67.66% (95% CI: 41.51–93.81%), *Proteus* spp.; 66.23% (95% CI: 34.65–97.82%), *Citrobacter* spp.; 63.78% (95% CI: 41.08–86.48%), *H. influenzae*; 62.50% (95% CI: 34.21–90.79%), *S. pneumoniae*; 62.45% (95% CI: 44.64–80.27%), and *S. aureus*; 61.71% (95% CI: 48.43–75.00%) (Supplementary Figure S3).

### Publication bias

**Figure 8:**
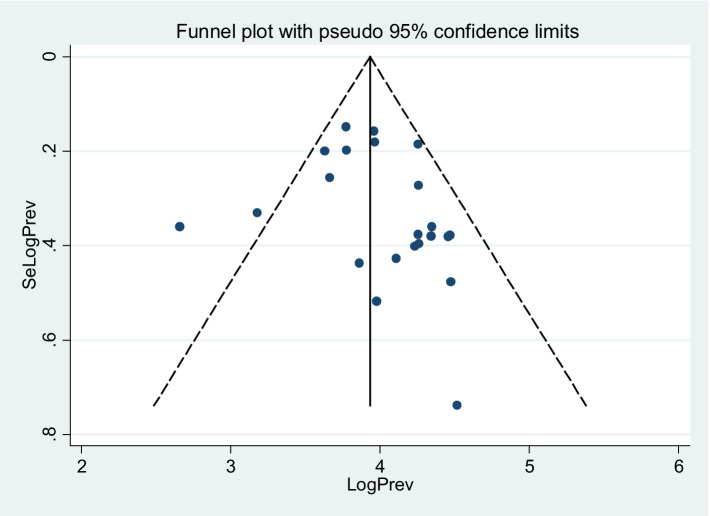
Funnel plot indicated publication bias in the studies on the co-trimoxazole resistance among HIV-infected individuals in Ethiopia.

Studies were assessed for potential publication bias with a subjective examination of funnel plot and statistics using Egger’s test and funnel plot (*p*-value = 0.028), indicating the presence of publication bias (Table 2; Figure 8).

**Table 2:** Publication bias using Egger’s test.

Egger’s test						
Std_Eff	Coef.	Std. Err.	*t*	*p* > |t|	[95% Conf. Interval]	
Slope	92.583	11.19163	8.27	0.000	69.15865	116.0073
Bias	−3.702535	1.618064	−2.29	0.034	−7.089181	−0.3158886

### Sensitivity analysis

**Figure 9:**
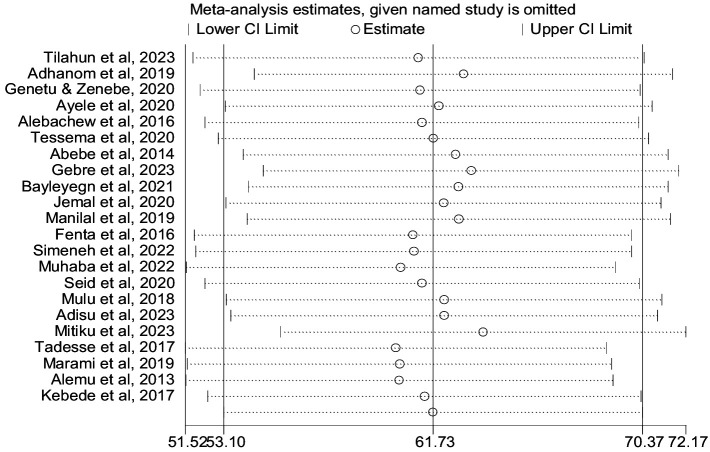
Sensitivity analysis.

We conducted a sensitivity analysis for the prevalence of co-trimoxazole resistance using a random effects model by step-by-step removal of each study. The results showed that the exclusion of studies does not have a significant effect on the pooled prevalence of co-trimoxazole resistance among HIV-infected individuals in Ethiopia (Figure 9).

## Discussion

This systematic review and meta-analysis is the first to determine the pooled prevalence of co-trimoxazole resistance in bacteria causing different types of infection or colonization among HIV-infected individuals in Ethiopia. The study included all available articles published from 2013 to 2023. Our study also determined the prevalence of co-trimoxazole resistance across the different regions in Ethiopia, the study period, sampling technique, types of infection or colonization, and bacterial group in the subgroup analysis to show whether there is a variation in the prevalence of co-trimoxazole resistance. In addition, this study excludes reports related to tuberculosis, sexually transmitted infections (viral and protozoa), and parasite-causing opportunistic infections in HIV-infected individuals. Moreover, this study provides information on co-trimoxazole resistance based on specific bacterial spp. in HIV patients.

According to recent studies from the African continent, Gram-negative pathogenic bacteria show significant rates of co-trimoxazole resistance, ranging from 50 to 97% (35–38). In this study, the pooled prevalence of co-trimoxazole resistance among HIV-infected individuals was 61.73% in all types of bacteria. In addition, the prevalence of co-trimoxazole resistance was higher in *E. coli*, followed by *Salmonella* and *Proteus* spp. This finding is consistent with a 50.3% Ethiopian report on the pooled prevalence of co-trimoxazole resistance in enteric bacteria among children (39). Another systematic review and meta-analysis in Iran revealed the prevalence of co-trimoxazole resistance among different uropathogenic bacterial spp. such as *E. coli*, *Klebsiella*, *Staphylococcus*, and *Enterobacter* (40). A meta-analysis by Olaru et al. also reported the resistance of co-trimoxazole to *S. aureus*, *S. pneumoniae, E. coli*, and *K. pneumoniae* that were identified in HIV-infected individuals (41).

It is suggested that co-trimoxazole prophylaxis may cause resistance in HIV-infected individuals for a variety of reasons, including prior exposure to co-trimoxazole or other sulphonamide-based drugs, non-adherence to co-trimoxazole prophylaxis, mutation, and patient self-medication, even though it lowers the risk of antimicrobial resistance by preventing infections and minimizing hospitalizations (42, 43). According to a study, there is evidence of cross-resistance between co-trimoxazole and penicillin antibiotics, and co-trimoxazole prophylaxis finally leads to the selection of co-trimoxazole resistance and MDR in pneumococci (15).

Additionally, co-trimoxazole is used for the prevention of *Pneumocystis jiroveci* (previously *carinii*) pneumonia and bacterial opportunistic infections in HIV-infected individuals. In this study, the subgroup analysis revealed a higher co-trimoxazole resistance in bacterial uropathogens, followed by bloodstream infection and pneumonia. Our finding is supported by a systematic review and meta-analysis that reported a high prevalence of co-trimoxazole resistance in uropathogens such as *E. coli*, *Klebsiella*, *Staphylococcus*, and *Enterobacter* (40). The selection of the appropriate antibiotics for treatment has become difficult and is primarily based on the information obtained from determining the pattern of antimicrobial resistance in the given area. This is because bacteria have acquired antibiotic resistance genes over time in different geographical areas and because there has been a change in the susceptibility patterns of bacteria to antibiotics (44).

Moreover, there was a significant heterogeneity among studies (I^2^ = 87.7%), which is not surprising given the variation in study settings, study period, type of bacterial infection or colonization, and type of bacterial species identified in the studies. A meta-analysis of co-trimoxazole resistance for most bacteria could not be performed because of the small number of studies reported, and we included bacteria reported in at least three studies. The presence of high heterogeneity across studies and limited number of studies in certain regions may impact the generalizability of findings.

### Limitations of the study

As a strength, this study is the first report of pooled prevalence of co-trimoxazole resistance among HIV-infected individuals in Ethiopia. Due to the absence of published articles, we could not include all regions in Ethiopia, which resulted lack of generalizability for the problem. In addition, viral load, CD4 count, and co-trimoxazole prophylaxis were not fully reported in our systematic review and meta-analysis.

## Conclusion and recommendations

According to our study, there is a higher prevalence of co-trimoxazole resistance to bacterial species causing opportunistic infections or colonization in HIV-infected individuals. This provides information for the WHO recommendation of co-trimoxazole prophylaxis to consider the need for modification to the prophylaxis agent and a detailed review of current guidelines. It can include medical care and regular follow-up to monitor compliance and side effects during co-trimoxazole administration. Moreover, epidemiologic surveillance on the extent of co-trimoxazole resistance is required in Ethiopia as a whole by including regions not described in this study.

## Data availability statement

The raw data supporting the conclusions of this article will be made available by the authors, without undue reservation.

## Author contributions

MA: Conceptualization, Data curation, Formal analysis, Methodology, Software, Validation, Visualization, Writing – original draft, Writing – review & editing. GG: Data curation, Formal analysis, Methodology, Writing – review & editing.
