# Supplementary material for: Prevalence of co-trimoxazole resistance among HIV-infected individuals in Ethiopia: a systematic review and meta-analysis
Source: Front Med (Lausanne). 2024 Jul 11;11:1418954. doi: 10.3389/fmed.2024.1418954 (PMC11285336; doi:10.3389/fmed.2024.1418954)
Supplement: Supplementary file 2 [file Table_2.DOCX]

**Table. S2 Quality assessment of the studies included in systematic review and meta-analysis on the prevalence of co-trimoxazole resistance among HIV-infected individuals in Ethiopia.**

| Author, year of publication | Q1 | Q2 | Q3 | Q4 | Q5 | Q6 | Q7 | Q8 | Q9 | Total score (9%) |
| --- | --- | --- | --- | --- | --- | --- | --- | --- | --- | --- |
| Tilahun et al, 2023 | Y | Y | Y | Y | Y | Y | Y | Y | Y | 9 |
| Adhanom et al, 2019 | Y | Y | N | Y | Y | Y | Y | Y | Y | 8 |
| Genetu & Zenebe, 2020 | Y | Y | Y | Y | Y | Y | Y | Y | N | 8 |
| Ayele et al, 2020 | Y | Y | N | Y | Y | Y | Y | Y | Y | 8 |
| Alebachew et al, 2016 | Y | Y | N | Y | Y | Y | Y | Y | NA | 7 |
| Tessema et al, 2020 | N | Y | Y | Y | Y | Y | Y | Y | Y | 8 |
| Abebe et al, 2014 | N | Y | N | Y | Y | Y | Y | Y | Y | 7 |
| Gebre et al, 2022 | Y | Y | Y | Y | Y | Y | Y | Y | NA | 8 |
| Bayleyegn et al, 2021 | Y | Y | Y | N | Y | Y | Y | Y | NA | 7 |
| Jemal et al, 2020 | Y | Y | Y | NA | Y | Y | Y | Y | Y | 7 |
| Manilal et al, 2019 | Y | Y | Y | Y | Y | Y | NA | Y | Y | 8 |
| Fenta et al, 2016 | Y | Y | Y | NA | Y | Y | Y | Y | Y | 8 |
| Simeneh et al, 2022 | Y | Y | Y | Y | Y | Y | NA | Y | NA | 7 |
| Muhaba et al, 2022 | Y | Y | NA | NA | Y | Y | Y | Y | Y | 7 |
| Seid et al, 2020 | Y | Y | Y | Y | Y | Y | Y | Y | Y | 9 |
| Mulu et al, 2018 | Y | Y | Y | Y | Y | Y | Y | Y | Y | 9 |
| Adisu et al, 2023 | Y | Y | Y | N | Y | Y | Y | Y | Y | 8 |
| Mitiku et al, 2023 | Y | Y | Y | N | Y | Y | Y | Y | Y | 8 |
| Tadesse et al, 2017 | Y | Y | Y | Y | Y | Y | Y | Y | Y | 9 |
| Marami et al, 2019 | Y | Y | Y | NA | Y | Y | Y | Y | Y | 8 |
| Alemu et al, 2013 | Y | Y | Y | NA | Y | Y | Y | Y | Y | 8 |
| Kebede et al, 2017 | Y | Y | Y | Y | Y | Y | Y | Y | Y | 9 |

**Key:** **Y** = Yes; **N** = Not reported, **NA** = Not appropriate

**Question codes:**

1. Was the sample frame appropriate to address the target population?

2. Were study participants sampled in an appropriate way?

3. Was the sample size adequate?

4. Were the study subjects and the setting described in detail?

5. Was the data analysis conducted with sufficient coverage of the identified sample?

6. Were valid methods used for the identification of the condition?

7. Was the condition measured in a standard, reliable way for all participants?

8. Was there appropriate statistical analysis?

9. Was the response rate adequate, and if not, was the low response rate managed appropriately?
