# Supplementary material for: Prevalence of co-trimoxazole resistance among HIV-infected individuals in Ethiopia: a systematic review and meta-analysis
Source: Front Med (Lausanne). 2024 Jul 11;11:1418954. doi: 10.3389/fmed.2024.1418954 (PMC11285336; doi:10.3389/fmed.2024.1418954)
Supplement: Supplementary file 3 [file Table_3.DOCX]

Fig. S3 **Forest plots showed the pooled prevalence of co-trimoxazole resistance in bacterial species among HIV patients in Ethiopia.**
